# Re-examining the role of hematopoietic stem cell transplantation in relapsed large B-cell lymphoma in the era of chimeric antigen receptor (CAR) T-cell therapy

**DOI:** 10.3389/fonc.2024.1397186

**Published:** 2024-08-15

**Authors:** Tamara K. Moyo, Rakhee Vaidya

**Affiliations:** ^1^ Department of Hematologic Oncology and Blood Disorders, Atrium Health Levine Cancer Institute, Charlotte, NC, United States; ^2^ Department of Hematology and Oncology, Atrium Health Wake Forest Baptist Comprehensive Cancer Center, Winston Salem, NC, United States

**Keywords:** relapsed large B-cell lymphoma (LBCL), CAR T-cell therapy, autologous stem cell transplant (ASCT), tisagenlecleucel, axicabtagene ciloleucel, lisocabtagene maraleucel

## Abstract

Historically, salvage chemoimmunotherapy with consolidative autologous hematopoietic stem cell transplantation (ASCT) was the only potentially curative therapeutic option for patients with relapsed/refractory large B-cell lymphoma (LBCL). Treatment options were few and outcomes poor for patients whose lymphoma failed to respond to salvage chemotherapy/ASCT and for patients not eligible for ASCT. The approval of chimeric antigen receptor (CAR) T-cell therapy for relapsed/refractory LBCL revolutionized the treatment landscape with unprecedented response rates and durability of responses. As a result, earlier intervention with CAR T-cell therapy has been explored, and the enthusiasm for CAR T-cell therapy has overshadowed ASCT. In this article, we will review the data available for ASCT and CAR T-cell therapy in relapsed LBCL and will examine the role for ASCT in relapsed/refractory LBCL in the era of CAR T-cell therapy.

## Introduction

Large B-cell lymphoma (LBCL) including *de novo* diffuse large B-cell lymphoma (DLBCL), primary mediastinal B-cell lymphoma (PMBCL), transformed indolent lymphoma, and high grade B-cell lymphoma (HGBL) have a typically aggressive course but are treatable and potentially curable types of B-cell non-Hodgkin lymphoma (B-NHL). The likelihood of achieving cure is variable and influenced by lymphoma characteristics such as histology and cytogenetics, by patient specific factors such as age, comorbidities and access to care, and by the first line treatment strategy employed. All current first line treatment regimens for LBCL include multi-agent anthracycline-based chemotherapy, a steroid and an anti-CD20 antibody. The addition of the anti-CD20 antibody rituximab to first line therapy for LBCL in the early 2000s had a major impact on response rates and overall survival (1–3). More recently, in the phase 3 POLARIX study replacing vincristine in the standard R-CHOP regimen (rituximab, cyclophosphamide, doxorubicin, vincristine and prednisone) with polatuzumab vedotin, an anti-CD79b antibody-drug conjugate, led to higher response rates in patients with advanced DLBCL (78% complete response rate in patients treated with pola-R-CHP vs. 74% with R-CHOP (4). Dose adjusted R-EPOCH (rituximab, etoposide, prednisone, vincristine, cyclophosphamide, and doxorubicin), a high intensity regimen with extended infusions and escalating doses of chemotherapy, did not improve outcomes in DLBCL patients in the Alliance/CALGB 50303 study (5) but is still employed for some subtypes of LBCL based on single arm prospective studies or retrospective data (6, 7).

Although many LBCL patients may be cured with first line therapy, many will relapse. 30-40% of DLBCL patients may be refractory to or relapse after first line treatment with the highest risk of relapse within the first 2 years (8). In a multi-center retrospective analysis of high grade B-cell lymphoma with *MYC* and *BCL2* rearrangements (so called double hit lymphoma), 2y PFS and OS after first line chemoimmunotherapy were 40 and 49%, respectively (7). For those LBCL patients who either do not respond to first line therapy or who relapse after an initial response, curative treatment options are limited, and the likelihood of cure is slim. In the SCHOLAR-1 pooled analysis of patients with refractory DLBCL, defined as progressive or stable disease as best response to any line of therapy or relapse within 1 year after ASCT, who were included in 4 clinical trial and observational cohort datasets, response rates to salvage chemotherapy were uniformly low with a pooled response rate 26% and a pooled complete response rate just 7%, underscoring the need for alternative therapies for patients in this group (9). In an analysis of patients with relapsed aggressive LBCL treated with high intensity regimens like R-EPOCH in the first line setting, the overall response rate to second line chemoimmunotherapy was 44% but the median PFS just 3 months and OS 8 months (10). Patients who relapsed in a later time frame after first line therapy did have improved outcomes (8, 11). In general, a short duration of first remission or failure to achieve remission have categorized patients into a high risk group.

The treatment landscape is evolving for LBCL patients who either do not respond to or who relapse after first line therapy. Historically, patients with relapsed LBCL have been treated with salvage chemoimmunotherapy. Responses to second line chemoimmunotherapy may be consolidated with high dose chemotherapy followed by an ASCT in fit patients. However, only a subset of patients with chemosensitive relapsed LBCL will achieve a durable response to salvage therapy. Thus, there was still an unmet need for improved salvage therapies, particularly for patients who relapsed early after an initial response to first line therapy or who had chemo-refractory disease. Chimeric antigen receptor (CAR) T-cell therapy was developed in this space and revolutionized the treatment landscape for relapsed LBCL, offering another potentially curative treatment option to patients including those with chemo-refractory disease. This article will review the evolution of the treatment paradigm for patients with relapsed/refractory LBCL and explore the role for ASCT in the modern era of cellular therapy.

## Salvage chemotherapy and ASCT in relapsed/refractory LBCL

In the pre-CAR T-cell era, the only potentially curative option for patients whose LBCL relapsed after anthracycline-containing induction chemotherapy was salvage chemotherapy followed by consolidative ASCT. Consolidative ASCT improves duration of remission, PFS, and OS in patients who respond to salvage chemotherapy. In the pre-rituximab era, the Parma group demonstrated a clear benefit of ASCT in patients who had chemo-sensitive relapsed B-NHL. In the Parma study, patients with relapsed B-NHL who had a response after 2 cycles of salvage DHAP (dexamethasone, cisplatin and cytarabine) were randomized either to receive an additional 4 cycles of DHAP + radiotherapy or to ASCT + radiotherapy. For those who responded to DHAP, outcomes were significantly better in the group who also received high dose chemotherapy and ASCT. At 5 years, both event free survival (EFS) and overall survival (OS) were significantly higher in the ASCT group compared to no ASCT (5y EFS: 46% vs 12%, 5y OS: 53% vs 32%) (12). As a result of this study, high dose chemotherapy and ASCT became standard of care for patients with relapsed LBCL that responded to second-line chemotherapy.

Multiple studies have shown that outcomes are similarly improved after ASCT in the rituximab era. The addition of rituximab to second-line therapy improved the likelihood of achieving a response which made ASCT more attainable (13). In the CORAL study, patients treated with rituximab-containing second-line therapy followed by high dose chemotherapy and ASCT had a 3 year PFS of 53% (14). In a retrospective European Blood and Marrow Transplantation registry study, 5 year disease free survival (DFS) after ASCT in the rituximab era was 48% (15). The more recent ORCHARRD study randomized patients with relapsed DLBCL to second line treatment with R-DHAP followed by ASCT or ofatumumab-DHAP (O-DHAP) followed by ASCT. There were no statistically significant differences in PFS, EFS or OS between the patients treated with R-DHAP or O-DHAP. Fewer than 40% of patients on either arm achieved a complete or partial response to second-line therapy and received ASCT per protocol. For those patients who did receive consolidative ASCT, outcomes were similar to those in PARMA and CORAL (2 year PFS and OS in the ORCHARRD study for patients treated with R-DHAP + ASCT were 52% and 68%, respectively). Highlighting the need for alternative non-chemotherapy salvage regimens, the 2y PFS and OS of patients treated with R-DHAP irrespective of transplant status were 26% and 38%, respectively) (16).

Duration of response to first-line therapy has consistently demonstrated an impact on outcomes after transplant. Patients with primary refractory LBCL or early relapse (e.g. relapse <12 months from diagnosis) are more likely to be chemo-refractory and have a relatively poor prognosis. In a multiregression analysis of prognostic factors predicting a response to DHAP salvage therapy in the Parma study, patients who relapsed > 12 months after diagnosis were nearly 3 times as likely to achieve a response (17). Regardless of whether they had relapsed early or late after first line therapy, the relative risk of progression was similarly increased in patients who responded to DHAP but who did not receive ASCT as compared to those who received ASCT. However, more than half of patients with early relapse who received ASCT after DHAP had progressed in the first year, whereas patients with late relapse had median PFS closer to 5 years (17). In the CORAL study patients who received rituximab-containing therapy and relapsed within 12 months of diagnosis had 3y PFS 39% with ASCT vs 14% without ASCT (14), providing further evidence that ASCT does improve outcomes regardless of time to relapse. However, the 3y PFS in the patients with early relapse still fell short of the 53% 3y PFS in all patients treated with ASCT on the study regardless of time to relapse (14). In both Parma and CORAL, ASCT improved PFS, but there was a significant gap in outcomes of patients with early relapse receiving ASCT compared to the entire group who received ASCT. The ORCHARRD study also demonstrated improved outcomes in patient with late relapse (>12 months from first-line therapy) as compared to patients who relapsed early after or who had suboptimal response to first-line therapy (16).

Response to therapy prior to ASCT and depth of response are important predictors of outcomes after ASCT in relapsed LBCL. Although ASCT may prolong the disease free interval, patients with active disease at the time of ASCT typically fail to achieve a cure. In an international multicenter study of patients with refractory LBCL, zero of 34 patients with refractory disease transplanted without ever having achieved remission were alive at 3 years (18). Patients who had an initial response to first line therapy but no response to second-line therapy had 3y DFS 14% after ASCT, whereas patients with chemo-sensitive lymphoma who achieved a response after both first and second line therapy had 3y DFS 30% after ASCT (18). Improved PFS was seen in patients who achieved complete response to rituximab containing salvage regimens prior to ASCT in the CORAL study as compared to patients who had only a partial response to therapy (14).

Multiple studies have demonstrated the prognostic importance of 18-fluorodeoxyglucose positron emission tomography (FDG-PET) after salvage chemotherapy but before ASCT in relapsed LBCL. In one study, only 3 of 30 patients with a negative FDG-PET prior to ASCT had relapsed with a median PFS of 1083 days, whereas only 4 patients with a positive FDG-PET prior to ASCT were still in remission with median PFS 402 days (19). In another study, median PFS of patients with a negative FDG-PET prior to ASCT was not reached vs 15.4 months in those with a positive FDG-PET (20). In an analysis of 129 patients with relapsed/refractory DLBCL, pre-ASCT FDG-PET response was the only pre-transplant risk factor that predicted both PFS and OS after ASCT. The 3y PFS and OS for patients with chemo-sensitive relapsed LBCL who achieved a complete metabolic response (Deauville score 1-3) on pre-transplant FDG-PET was 77% and 86% respectively versus 49% and 54% respectively in patients with partial response (Deauville 4) on FDG-PET prior to ASCT (21). Patients in the ORCHARRD study who had a complete response based on FDG-PET imaging after 3 cycles of salvage R-DHAP/O-DHAP also had significantly improved PFS/OS after ASCT than patients who had a positive FDG-PET scan (16).

Although consolidative ASCT has demonstrated improved outcomes in patients who respond to salvage chemoimmunotherapy, it is important to consider that many patients do not respond to salvage therapy or are not candidates for ASCT. In the Parma study, more than 40% of patients did not achieve a response to salvage DHAP and were not eligible for ASCT (12). Although many studies have shown that it is feasible to transplant elderly patients with relapsed lymphoma (22–24), the data are challenging to interpret in large part due to the retrospective nature of the studies with inherent selection bias and variable definitions of “elderly”. Perhaps more important than age, patient comorbidities may impact the risk of non-relapse mortality with ASCT (25). Patients typically must undergo rigorous testing to ensure fitness for ASCT. For patients who have no response or only partial response to salvage therapy or who may be unfit for transplantation due to comorbidities, a more accessible approach with similarly improved chance to achieve disease control/cure was lacking until the advent of CAR T-cell therapy.

## CAR T-cell therapy in relapsed LBCL

CAR T-cell therapy was developed to address the unmet need for effective therapies with durable response in patients with relapsed/refractory LBCL. A detailed description of CAR T-cell features and manufacturing is beyond the scope of this review article. Simply put, autologous CAR T-cell therapy for lymphoma involves the collection of a patient’s own T-cells through leukapheresis and genetic modification of the T-cells to express a chimeric receptor with coactivation domains that home the activated T cells to the lymphoma upon reinfusion into the patient. All currently approved CAR T-cell products for LBCL target the CD19 protein on the surface of the lymphoma cells, although other targets are actively under investigation. Lymphodepletion chemotherapy (LDC), typically incorporating fludarabine and cyclophosphamide, is given prior to CAR T-cell therapy to create a more hospitable environment for CAR T-cells to flourish and proliferate.

There are now three approved autologous CAR T-cell products for relapsed/refractory LBCL available in the United States. Axicabtagene ciloleucel (axi-cel) was the first approved CD-19 directed CAR T-cell therapy for relapsed/refractory LBCL after two prior lines of therapy. In the ZUMA-1 phase 2 clinical trial, axi-cel was administered to 111 patients with refractory DLBCL, PMBCL, or transformed follicular lymphoma (tFL). Response rates were unprecedented, with an 82% objective response rate (ORR) and 54% complete response (CR) rate (26). After a median follow-up of 63 months, median duration of response to axi-cel was 11.1 months but 31% of patients had ongoing response at 5 years (27). In the TRANSCEND NHL 001 study, 269 patients with relapsed or refractory LBCL were treated with lisocabtagene maraleucel (liso-cel). At a median follow-up of 19.9 months, the ORR and CR rate to liso-cel were 73% and 53% respectively. The median duration of response to liso-cel was 23.1 months (28). In the JULIET study, after a median follow-up of 40 months, ORR and CR rate in 93 patients treated with tisagenlecleucel (tisa-cel) were 53% and 39% respectively (29). As a result of these studies, axi-cel, liso-cel, and tisa-cel received initial regulatory approvals for relapsed/refractory LBCL after two prior lines of therapy. Real world analyses have largely confirmed the responses to CAR T-cell therapy seen in the registrational clinical trials.

Whereas side effects of consolidative high dose chemotherapy followed by ASCT are similar to side effects typical of salvage chemoimmunotherapy, CAR T-cell therapy has a unique side effect profile. Acute side effects of CAR T-cell therapy include cytokine release syndrome (CRS) and immune effector cell-associated neurotoxicity syndrome (ICANS), but patients receiving CAR T-cell therapy may also experience prolonged cytopenias, hypogammaglobulinemia, and increased risk of infections. The risk of side effects varies widely amongst the three CAR T-cell products. Westin, et al. presented a comparison of safety and efficacy of the three commercially available anti-CD19 CAR T-cell products in the ZUMA-1, TRANSCEND and JULIET trials, with rates of any grade CRS ranging from 42% - 92% and of any grade ICANS 21% - 67%. The majority of CRS and ICANS events were grade 1/2, regardless of the product (30).

Although adverse events due to CAR T-cell therapy are common, the majority are low grade and manageable with conventional means including tocilizumab for CRS or steroids for ICANS. Thus, elderly or unfit patients are not excluded from CAR T-cell therapy. Patients > 70 years treated with CAR T-cell therapy had similar kinetics of T cell expansion, similar rates of grade ≥ 3 CRS or ICANS, and similar outcomes as younger patients undergoing the same therapy. Objective response rate was 63% in patients > 70 years and CR rate 46% (31). PFS and OS at 1 year were 32% and 69% respectively (31). Although poor performance status and comorbidities did predict for inferior survival after CAR T-cell therapy, presence of comorbidities was not associated with incidence of CRS, ICANS or admission to ICU (32).

In addition to the distinct side effects of CRS and ICANS, there are shared side effects between ASCT and CAR T-cell therapy. The risk of cytopenias after CAR T-cell therapy is variable and influenced by the lymphodepletion chemotherapy, the CAR T-cell product, inflammatory responses to CAR T-cell therapy (e.g. severity of CRS or immune-effector cell-associated hemophagocytic syndrome or IEC-HS), presence of infections, disease burden, among others. In a subset of patients, cytopenias may be severe and/or prolonged. Up to 16% of patients remained neutropenic and up to 38% thrombocytopenic more than 3 months after CAR T-cell infusion on the ZUMA-1, TRANSCEND and JULIET trials (26, 28, 29). For comparison, in one large retrospective study including 1182 patients who received ASCT, none had neutropenia persisting beyond 30 days, whereas 9.6% had thrombocytopenia beyond 90 days after ASCT (33). Bacterial and viral infections are also common after both ASCT and CAR T-cell therapy, with the risk increased in patients with a history of infections prior to CAR T-cell therapy and in patients treated with corticosteroids for CRS or ICANS (34). Finally, the incidence of second primary malignancy after CAR T-cell therapy is an area of particular interest after the FDA released a report of secondary T-cell neoplasms (35). A large multi-center retrospective study of 582 patients who received CAR T-cell therapy for relapsed LBCL, identified 45 cases (8.2%) with second primary malignancy, the most common of which was myelodysplastic syndrome, diagnosed at a median of 19.3 months after CAR T-cell infusion (36). The incidence of second primary malignancy after ASCT is estimated to be 10-15% at 15 years after ASCT (37, 38).

The impact of tumor burden on CAR T-cell efficacy has also been explored in retrospective analyses. Although the registrational studies required patients undergoing CAR T-cell therapy to have measurable disease, retrospective real-world analyses have shown that CAR T-cell therapy may perform better with lower tumor burden. Patients with low metabolic tumor volume treated with axi-cel for relapsed/refractory LBCL had improved OS and PFS as compared to patients with high metabolic tumor volume (39). Wudhikarn et al. reported good outcomes for patients who had no residual lymphoma at the time of CAR T-cell therapy. At 1 year post CAR T-cell therapy, only 39.4% of patients had relapsed and OS was 81.3% (40). In fact optimal bridging therapy may reduce the risk of disease progression or death by as much as 40% after CAR T-cell therapy (41). Radiation has been explored as bridging therapy and was found to effectively cytoreduce bulky tumors, lower LDH, and reduce metabolic tumor volume, all of which have been associated with poor responses to CAR T-cell therapy (42, 43). Importantly, bridging radiation had no adverse impact on safety outcomes.

## CAR T-cell therapy versus ASCT in relapsed LBCL

Given the unprecedented responses to CAR T-cell therapy in patients with relapsed/refractory LBCL after at least 2 lines of therapy, attention turned to optimizing the risk/benefit ratio of CAR T-cell therapy through a variety of mechanisms. One consideration was whether the efficacy could be improved and risks reduced by employing CAR T-cell therapy earlier in the treatment paradigm for LBCL. Prospective clinical trials were launched that randomized LBCL patients in first relapse to receive standard of care therapy with second line chemoimmunotherapy followed by consolidative ASCT in patients with chemo-sensitive relapse or a commercially available CAR T-cell product. Salvage chemotherapy plus ASCT was compared to axi-cel in the ZUMA-7 clinical trial, to liso-cel in the TRANSFORM study, and to tisa-cel in the BELINDA clinical trial (**Table 1**).

**Table 1:** Pivotal trials comparing CAR T-cell therapy and Standard of Care in first relapse.

	ZUMA-7^a^		TRANSFORM^b^		BELINDA^c^	
	Axi-cel N=180	SOC N=179	Liso-cel N=92	SOC N=92	Tisa-cel N=162	SOC N=160
Patient characteristics						
*Median age, years (range)*	58 (21 - 80)	60 (26-81)	60 (20 - 74)	58 (26 - 75)	59.5 (19 - 79)	58 (19 - 77)
*Age ≥ 65 years, no. (%)*	51 (28)	58 (32)	36 (39)	25 (27)	54 (33.3)	46 (28.8)
*Male sex, no. (%)*	110 (61)	127 (71)	44 (48)	61 (66)	103 (63.6)	98 (61.2)
*White, no. (%)*	145 (81)	152 (85)	Not reported	Not reported	128 (79)	128 (80)
*AA-IPI ≥2^a, b^ or IPI ≥2 ^c^, no. (%)*	82 (46)	79 (44)	36 (39)	37 (40)	106 (65.4)	92 (57.5)
*ECOG PS 1, no. (%)*	85 (47)	79 (44)	44 (48)	35 (38)	70 (43.2)	65 (40.6)
Clinical characteristics						
*Stage III or IV, no. (%)*	139 (77)	146 (82)	68 (74)	63 (68)	107 (66.0)	98 (61.3)
*High grade B-cell lymphoma, no. (%)*	31 (17)	26 (15)	22 (24)	21 (23)	39 (24.1)	27 (16.9)
*Double expressor lymphoma, no. (%)*	57 (32)	62 (35)	Not reported	Not reported	Not reported	Not reported
*Relapse at ≤12 months after first line therapy, no. (%)*	47 (26)	48 (27)	25 (27)	22 (24)	55 (34.0)	53 (33.1)
*Refractory to first-line therapy, no. (%)*	133 (74)	131 (73)	67 (73)	70 (76)	107 (66.0)	107 (66.9)
*Received bridging therapy, no. (%)*	65 (36)	---	58(63)	---	135 (83.3)	---
*Type of bridging therapy allowed*	glucocorticoids	---	platinum based therapy (1 cycle)	---	platinum based therapy	---
*Received CAR-T or ASCT, per protocol, no. (%)*	170 (94)	64 (36)	89 (97)	43 (47)	155 (97.5)	52 (32.5)
*Received CAR-T crossover, no. (%)*	---	N/A	---	58 (63)	---	81 (50.6)
Efficacy						
*Median EFS, months (95% CI)*	8.3 (4.5 - 15.8)	2.0 (1.6 - 2.8)	NR (9.5 - NR)	2.4 (2.2 - 4.9)	3.0 (3.0 - 3.5)	3.0 (2.9 - 4.2)
*Estimated 2-year^a^ or 18-month^b^ EFS, % (95% CI)*	41 (33 - 48)	16 (11 - 22)	52.6 (42.3 - 62.9)	20.8 (12.2 - 29.5)	Not reported	Not reported
*CR, no. (%)*	110 (61)	61 (34)	68 (74)	40 (43)	46 (28.4)	44 (27.5)
*Median OS, months (95% CI)*	NR (28.3 - NE)	35.1 (18.5 - NE)	NR (29.5 - NR)	29.9 (17.9 - NR)	16.9 (11.1 - NE)	15.3 (12.3 - NE)
*Median PFS, months (95% CI)*	14.7 (5.4 - NE)	3.7 (2.9 – 5.3)	NR (12.6 - NR)	6.2 (4.3 - 8.6)	Not reported	Not reported
*Estimated 2-year^a^ or 18-month^b^ PFS, % (95% CI)*	46 (38 - 53)	27 (20 - 35)	58.2 (47.7 - 68.7)	28.8 (17.7 - 40.0)	Not reported	Not reported
Safety						
*Grade ≥3 Adverse Event, no. (%)*	155 (91)	140 (83)	85 (92)	81 (89)	136 (84)	144 (90)
*Grade ≥3 Neutropenia, no. (%)*	118 (69)	69 (41)	75 (82)	47 (52)	65 (40.1)	63 (39.4)
*Grade ≥3 Thrombocytopenia, no. (%)*	25 (15)	95 (57)	46 (50)	62 (68)	52 (32.1)	76 (47.5)
*Any Grade ≥3 prolonged cytopenia, no. (%)*	49 (29)	12 (19)	40 (43)	3 (3)	Not reported	Not reported
*Grade ≥3 Febrile Neutropenia, no. (%)*	4 (2)	46 (27)	11 (12)	21 (23)	21 (13.0)	40 (25.0)
*Grade ≥3 CRS, no. (%)*	11 (6)	---	1 (1)	---	8 (5.2)	---
*Grade ≥3 Neurologic Event, no. (%)*	36 (21)	1 (1)	4 (4)	Not reported	3 (1.9)	Not reported

AA-IPI age adjusted international prognostic index; ASCT autologous stem cell transplant; Axi-cel axicabtagene ciloleucel; CAR-T chimeric antigen receptor T-cell therapy; CI confidence interval; CR complete response; CRS cytokine release syndrome; ECOG PS Eastern Cooperative Oncology Group Performance Status; EFS event free survival; IPI international prognostic index; Liso-cel lisocabtagene maraleucel; NE not estimable; NR not reached; OS overall survival; PFS progression free survival; SOC standard of care (e.g. salvage chemotherapy +/- ASCT); Tisa-cel tisagenlecleucel.

a ZUMA-7 (44).

b TRANSFORM (45).

c BELINDA (46).

In the phase 3 ZUMA-7 study, 180 patients with primary refractory/early relapsed LBCL were randomized to receive axi-cel, and 179 patients to receive salvage chemoimmunotherapy plus ASCT as per standard of care (SOC). Bridging chemotherapy was not allowed in the axi-cel arm. Notably drop-out in the SOC arm was high, mostly due to lack of response to salvage chemotherapy, with only 64 (36%) randomized patients receiving salvage chemotherapy and ASCT per protocol as compared to 96% of patients randomized to the axi-cel arm who received the infusion (44). Median progression free survival in the axi-cel arm was 14.7 months and 3.7 months in the SOC arm. Axi-cel was favored as second line treatment overall (HR 0.40, 95% CI 0.31-0.51). There were no subgroups in which SOC was favored (44). Despite that more than 50% of patients treated on the SOC arm went on to receive cellular immunotherapy off protocol, there was improved overall survival in the axi-cel arm (median not reached vs 35.1 months in the SOC arm), although the difference was not statistically significant. Interestingly, a naïve T-cell phenotype (CCR7^+^CD45RA^+^) thought to represent stem memory T-cells was more prevalent in patients treated with axi-cel in ZUMA-7 as compared to ZUMA-1 and were associated with improved progression free survival and duration of response but not toxicity, providing a plausible scientific explanation for the improved PFS in the ZUMA-7 trial and suggesting that T-cells may be more effective if collected and engineered to become axi-cel earlier in the treatment algorithm for relapsed LBCL (47).

In the phase 3 TRANSFORM study, patients with primary refractory or early relapsed LBCL were randomized to receive liso-cel or SOC with salvage chemo-immunotherapy and ASCT if chemo-sensitive. Bridging therapy was common (63%) in the liso-cel arm. Nearly 46% of patients randomized to SOC did receive ASCT. At a median follow-up of 17.5 months, median EFS was significantly prolonged in the liso-cel group (not reached versus 2.4 months in the SOC arm). Differences in median overall survival favored liso-cel but did not reach statistical significance (mOS not reached with liso-cel versus 6.2 months in the SOC arm; HR = 0.724; p 0.0987)) (45).

In the phase 3 BELINDA study, patients with refractory or early relapsed LBCL were randomized to receive tisa-cel (n= 162) or SOC chemoimmunotherapy and ASCT (n=160). There were important differences in the treatment groups, with a higher percentage of patients with HGBL and high IPI scores in the tisa-cel group. A majority of patients randomized to receive tisa-cel received bridging therapy (83%). Notably, patients treated on the SOC arm had response assessed after 6 weeks of therapy and if response was inadequate, they could receive another SOC therapy prior to ASCT. 54% of patients in the SOC arm did in fact receive more than one SOC salvage therapy, and yet only 32.5% went on to receive ASCT. The ORR and CR rates were not statistically different in the tisa-cel or SOC therapy arms. Likewise median survival was not statistically different in the two arms (median EFS 3.0 months in both groups and median OS 16.9 months in the tisa-cel arm vs 15.3 months in the SOC arm) (46). The results of the BELINDA trial did not support use of tisa-cel in the second line setting, potentially because of the inclusion of patients with higher risk in the tisa-cel arm or because manufacturing time was significantly longer than with the other two anti-CD19 CAR T-cell products, allowing more time for relapse to occur before tisa-cel infusion.

Despite the disappointing results in the BELINDA trial, the prospective ZUMA-7 and TRANSFORM studies underscore the potential for axi-cel and liso-cel in a high risk group of patients with LBCL enriched for chemo-refractory lymphoma and with expectedly poor outcomes with ASCT. As a result of these studies, axi-cel and liso-cel have received approval for treatment of LBCL patients in the second line who are either refractory to first line therapy or who relapse early (within 12 months of first-line therapy). Additionally, liso-cel is approved for second line therapy in patients who are not transplant-eligible based on age or comorbidities, regardless of timing of relapse with respect to their first line therapy.

Recent registry database studies may re-kindle the fire for ASCT in patients with chemo-sensitive relapsed LBCL. One study included patients with relapsed LBCL in partial response at the time of ASCT or CAR T included in the Center for International Blood & Marrow Transplant Research (CIBMTR) registry database. There were lower relapse rates and improved 2y OS in the ASCT group as compared to the axi-cel group (relapse rate 40% with ASCT vs 53% with axi-cel; 2y OS 69% for the patients who received ASCT vs 47% for patients who received axi-cel) (48). Similarly for patients with relapsed LBCL in the CIBMTR database who received additional chemoimmunotherapy and achieved a complete remission prior to CAR T-cell therapy or ASCT, outcomes were better for patients whose response was consolidated with ASCT as compared to CAR T-cell therapy. Relapse rate and OS at 2 years were 48% and 65.6% respectively in patients treated with CAR T-cell therapy as compared to 27.8% and 78.9% respectively in the ASCT group (49). Higher 2y relapse rate and inferior 2y PFS were also seen in the subgroup of patients with early relapse who achieved CR prior to CAR T-cell therapy as compared to ASCT (relapse rate 45.9% in CAR T-cell cohort vs 22.8% in ASCT; 2y PFS 48.3% after CAR T-cell therapy vs 70.9% after ASCT) (49). Although intriguing, the majority of patients treated with CAR T-cell therapy in this study received tisa-cel, which in both the JULIET and BELINDA studies had less favorable outcomes after CAR T-cell therapy as compared to outcomes of axi-cel or liso-cel in their respective phase 3 studies. The applicability of these findings at centers where the preferred CAR T-cell product is other than tisa-cel is uncertain. Prospective studies will be necessary to fully elucidate the optimal strategy for sequencing transplant and cellular therapy in patients with relapsed LBCL, especially those who are not at the extreme ends of relapse risk.

## Discussion

For decades, the only potentially curative option for patients with relapsed DLBCL was ASCT which was associated with 5-year OS of approximately 50% (12). Unfortunately, two thirds of patients with relapsed DLBCL were not candidates for ASCT due to advanced age, comorbidities, or chemo refractory disease. Long term outcomes in these patients was exceedingly poor. The availability of CAR T-cell therapy drastically improved outcomes in these patients, with more than 30% of patients achieving a sustained and durable remission after a single infusion of CD19-directed CAR T-cells. These impressive results in the third line (and beyond) setting led to randomized studies comparing CAR T-cell therapy to ASCT in second line. Results for two of the 3 randomized studies showed significantly improved outcomes with CAR-T in the second line as compared to salvage chemoimmunotherapy followed by ASCT. Notably, only one third of the patients who were randomized to the ASCT arm received ASCT; this is because a high proportion of patients had chemo refractory disease after salvage chemotherapy and were eventually treated with CAR T-cells off protocol.

These data clearly highlight the superiority of CAR T-cell therapy over ASCT in patients who have primary refractory disease or early relapse after front line chemoimmunotherapy. A significant number of these patients have high risk disease characteristics such as double hit lymphoma or high grade B-cell lymphoma intermediate between DLBCL and Burkitt. Our consensus for this group of patients is to proceed with CAR T-cell therapy since additional cytotoxic chemotherapy is unlikely to achieve long term disease control.

Another group of patients where CAR T-cell therapy is clearly superior to ASCT is older patients who are not candidates for ASCT due to frailty or other medical comorbidities. Real-world data from CAR T-cell therapy shows that patients who are older and have co-morbidities have similar outcomes after CAR T-cell therapy as those who were treated on the initial pivotal CAR-T clinical trials which had stringent inclusion and exclusion criteria (31, 50). The toxicities of CAR T-cell therapy including CRS and neurotoxicity can often be successfully treated with early recognition, prompt escalation of care, and medications such as tocilizumab and steroids. On the contrary, ASCT carries significantly higher toxicity due to the intensity of conditioning therapy which causes a significant period of cytopenias, mucosal toxicity, and risk of infection. Thus, our consensus is to proceed with CAR T-cell therapy for patients who are not candidates for ASCT due to older age, frailty, or co-morbidities, regardless of the timing of relapse after front line chemoimmunotherapy.

While the role of ASCT in treatment of relapsed DLBCL has significantly declined after availability of CAR T, there is still a sub group of patients where ASCT may be superior to CAR T-cell therapy. This subset includes patients who have disease relapse in a later timeframe after front line therapy and who are fit for ASCT. In this group of patients, it is reasonable to discuss pros and cons of ASCT and consider two to three cycles of platinum-containing salvage chemotherapy. Approximately half of the patients receiving salvage chemotherapy will have chemo refractory disease and will not be able to proceed with ASCT, eventually requiring CAR T-cell therapy. However, based on retrospective data from CIBMTR, a proportion of patients who achieve a complete or partial response to salvage chemotherapy may have better outcomes with ASCT as compared to CAR T-cell therapy (49). Similarly for patients who have already received second line chemoimmunotherapy prior to their referral for cellular therapy, the CIBMTR data would support proceeding to ASCT if they have achieved an optimal response. If relapse occurs after ASCT in these patients, the efficacy of CAR T-cell therapy in later lines of therapy is well-established with 5 year PFS of approximately 40%.

There are additional factors which could impact the decision making process around sequencing of therapies for patients with relapsed/refractory LBCL. While the efficacy of CAR T-cell therapy is well-established in patients who fail salvage chemotherapy and ASCT, it is unknown if a patient who failed second line CAR T-cell therapy might benefit from consolidation with ASCT after later lines of therapy or if autologous stem cell collection would even be feasible after CAR T-cell therapy. The advent of newer therapies may force us to re-examine the role of CAR T-cell therapy and sequencing of therapies for relapsed/refractory LBCL in the future. As an example, two CD20 x CD3 bispecific antibodies epcoritamab and glofitamab have been approved for use in relapsed LBCL, eliciting durable responses with lower rates of CRS or neurotoxicity as compared to CAR T-cell therapy, although no prospective studies have directly compared bispecific antibodies to CAR T-cell therapy. The incorporation of bispecific antibodies into earlier lines of therapy is an area of active investigation. It is unknown if bispecific antibody therapy could lead to T cell exhaustion or alteration of the T cell milieu in a way that could impact the manufacturing or efficacy of CAR T-cell therapy.

**Figure 1:**
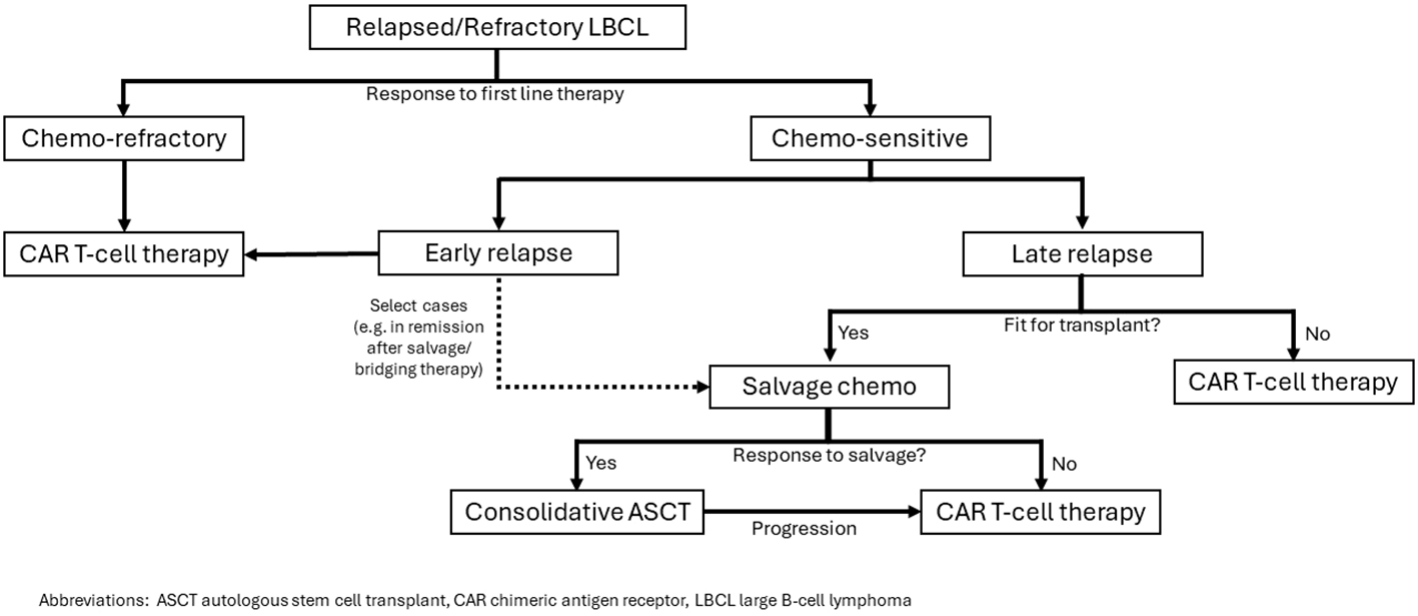
Our approach to selection of CAR T-cell therapy or ASCT for patients with relapsed/refractory LBCL.

In summary, CAR T-cell therapy has superseded ASCT in the treatment of relapsed DLBCL for a vast majority of patients. A small subset of young, fit patients who have a late relapse and chemotherapy sensitive disease may still have better outcomes with ASCT. Our approach (**Figure 1**) is to consider CAR T-cell therapy in patients who fail to achieve CR during first line of therapy or patients who relapse and are unfit for ASCT. For fit patients with late relapse, salvage chemoimmunotherapy followed by consolidative ASCT is favored. For patients fit for transplant who have an initial response to first line chemotherapy but relapse early, a careful weighing of the pros and cons of each approach may be warranted. Proceeding directly to CAR T-cell therapy with axi-cel or liso-cel would be supported by the ZUMA-7 and TRANSFORM studies. However, if the patient achieved a complete response to bridging chemoimmunotherapy, consolidation with ASCT and reserving CAR T-cell therapy for later relapse could be considered and supported by the CIBMTR data analyses.
